# Single-Metal-Atom Chains at Mirror Twin Boundaries in Transition Metal Dichalcogenides: Electronic, Magnetic, and Catalytic Properties

**DOI:** 10.1021/acsami.6c03473

**Published:** 2026-06-04

**Authors:** Prosun Santra, Mahdi Ghorbani-Asl, Wouter Jolie, Arkady V. Krasheninnikov

**Affiliations:** † Institute of Ion Beam Physics and Materials Research, 28414 Helmholtz-Zentrum Dresden-Rossendorf, 01328 Dresden, Germany; ‡ II. Physikalisches Institut, 14309 Universität zu Köln, Zülpicher Straße 77, 50937 Köln, Germany

**Keywords:** single-metal-atom chains, mirror twin boundaries, transition metal dichalcogenides, DFT calculations, electronic properties, magnetism, hydrogen evolution reaction

## Abstract

The experimentally realized atomic chains composed of transition metal atoms embedded into mirror twin boundaries (MTBs) in two-dimensional MoS_2_ represent a class of quasi-one-dimensional systems with intriguing magnetic and electronic characteristics. By employing first-principles calculations, we investigate the possibility of creating single-metal-atom chains (SMACs) in other Mo- and W-based H-phase transition metal dichalcogenides. Our results indicate that for the transition metal atoms with the number of d-electrons different from that in Mo and W, the formation of SMACs is energetically preferable over having isolated substitutional impurities in the basal plane of the TMDs, pointing out that these structures can be synthesized. We further study the magnetic and electronic properties of these systems and demonstrate that many structures exhibit half-metallic electronic behavior associated with a Peierls-like distortion and thus can be employed in spintronic applications. Finally, we investigate the potential of various SMACs to be used as a catalyst in the hydrogen evolution reaction (HER). Our calculations predict that some of the SMACs should exhibit HER activity comparable to or even superior to that of the standard Pt(111) surface.

## Graphical abstract

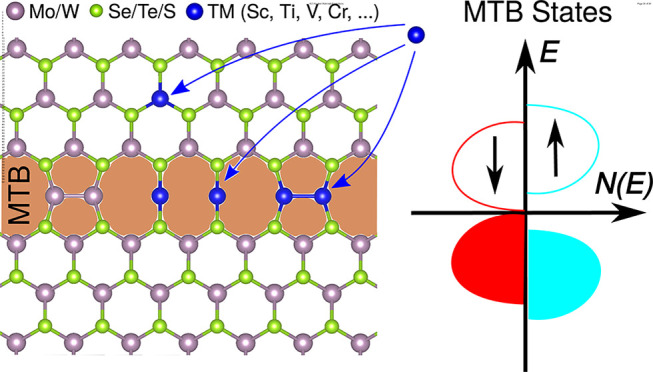

## Introduction

Materials with reduced dimensionality, and specifically one-dimensional (1D) metallic nanostructures, are of particular interest in condensed matter physics and materials science, as their confined geometry and strong electron correlations give rise to quantum phenomena that are absent in higher dimensions. These include the Peierls instability, which drives a metal-to-insulator transition via lattice distortion,^1–5^ non-Fermi liquid behavior described by the Tomonaga-Luttinger liquid (TLL) theory,^6–10^ and the emergence of exotic quasiparticles such as Majorana Fermions, which are of significant interest for topological quantum computing.^11,12^

The electronic and magnetic properties of these materials are equally compelling,^13–16^ making them interesting in the context of spintronics.^17^ Their versatile electronic characteristics,^9–12,18–20^ including phenomena such as giant magnetoresistance,^21–23^ hint also at potential applications in electronics and information storage.

However, the synthesis, stability, and scalability of well-defined 1D structures present considerable obstacles. A diverse arsenal of techniques has been developed to overcome this challenge. These methods range from physical approaches, such as mechanical break of junctions between macroscopic leads^24–26^ and manipulating individual atoms with the scanning probe microscope,^13,15,19^ to bottom-up growth of atomic chains via self-organization on crystalline substrates^18,27^ or at the edges of two-dimensional (2D) materials.^28^ Additional approaches involve confining metal chains within the hollow cores of carbon nanotubes^29–31^ or engineering 1D systems with cold trapped ions in optical lattices.^32^

Each fabrication technique faces its own distinct set of challenges related to stabilization of the chains and their characterization. For instance, the presence of an encapsulating material, e.g., a metallic carbon nanotube,^33^ can screen interactions and prevent the use of specific experimental methods for studying the intrinsic electronic properties of the chains inside the tube.^34^

A particularly promising route involves creating quasi-1D systems at the interfaces of 2D semiconductors, as on the one hand, the chains are stabilized by the atomic environment, and on the other, their characteristics can directly be assessed using various techniques, such as scanning tunneling microscopy (STM). This has successfully been demonstrated at the interfaces between different transition metal dichalcogenides (TMDs)^35^ and at the mirror twin boundaries (MTBs) in various TMDs.^36^ The weakly screened environment also leads to various many-body phenomena, such as Tomonaga-Luttinger liquids,^8^ Hubbard-type Coulomb blockade effects,^37^ or Kondo physics.^38^

Incorporating foreign atoms inside MTBs leads to chains with tailored electronic and magnetic properties. It has been shown by Guo et al.^39^ that embedding transition metal (TM) atoms into the MTBs of MoS_2_ results in the formation of 1D single-metal-atom chains (SMACs). Using a chemical vapor codeposition method, Pt SMACs at MTBs in MoS_2_ monolayers were grown, which were stable under ambient conditions with an average length of up to 17 nm. These metallic Pt chains formed an interconnected conduction pathway throughout the 2D film. Subsequent work by Qin et al.^40^ demonstrated the successful synthesis of Co-, Ni-, Pd-, and Pt-based SMACs within MoS_2_ using essentially the same technique. The creation of SMACs in MoS_2_ raises a fundamental question of whether this approach can be generalized to other TMD hosts in the H-phase and what the magnetic and electronic properties of these systems could be.

In this study, we seek to answer these questions. We present a systematic first-principles study of 27 different transition metal elements forming SMACs at the MTBs in four TMDs: MoSe_2_, MoTe_2_, WS_2_, and WSe_2_. For the sake of completeness, we also include previously published data^41^ for MoS_2_. To evaluate the potentials for the experimental realization of SMACs in these hosts, we assess the energetics of the configurations with TM atoms embedded in an MTB as compared to its position in the basal plane of TMDs, the approach we used previously^41^ and which predicted the high stability of the Ni and Pd SMACs, which were experimentally synthesized later on.^40^ Our calculations reveal that a subset of these systems exhibit half-metallicity and host magnetic states that are localized specifically at the SMACs, highlighting their potential for nanoscale spintronic devices.

In addition, as TMDs are currently under scrutiny in the context of catalysis as potential substitutes for platinum group metals to reduce catalyst cost,^42–45^ we further investigate the catalytic properties of various SMACs. Specifically, we study the hydrogen evolution reaction (HER) performance for different SMACs in MoSe_2_ and compare it to that of pristine/defective MoSe_2_ and Pt(111) surface. Our calculations show that some of the SMACs may exhibit HER activity comparable or even superior to that of the standard Pt(111) surface.

## Computational Approach

In our calculations, we used plane-wave density functional theory (DFT) with an energy cutoff of 500 eV. The calculations were performed using the Vienna Ab initio Simulation Package (VASP),^46^ employing the PBE functional^47^ to describe exchange and correlations and the projector augmented-wave (PAW) potentials.^48^ All calculations were spin-polarized, and spin–orbit coupling (SOC) was also taken into account in the calculations of the SMAC electronic structures.

For Brillouin zone integration, we used Monkhorst–Pack grids^49^ with a *k*-point density equivalent to an 8 × 8 × 1 grid for 2D supercells and an 8 × 1 × 1 grid for 1D structures, or higher. Supercells comprising 130 atoms were used, each containing two embedded transition metal (TM) atoms along the MTB direction. For exchange interaction calculations, we used supercells that were doubled in size, each containing four embedded TM atoms. A vacuum gap of 20 Å was implemented to mitigate spurious interactions arising from periodic boundary conditions. We note that due to the symmetry of the TMD ribbons with MTBs, the system can be made periodic only in one direction (along the SMAC), so that vacuum was added at the edges of the ribbons and in the out-of-plane directions; see ref 41 for details.

## Results and Discussion

### Energetics of Transition Metal Atom Chains at Mirror Twin Boundaries

Following the methodology we used in our previous work,^41^ to assess the feasibility to form a SMAC at the MTB for a TM atom A, we computed the energy difference Δ*E*_f_ between two configurations: the A atoms substituting for the Mo atoms at MTB in MoSe_2_, MoTe_2_ (for the W atom for WS_2_, WSe_2_), and for the metal atoms within the basal plane of the corresponding host material. The former configuration is denoted “C-sub” and its energy is calculated as follows:

1 $$E_{f}\left[\mathrm{C‐}\mathrm{sub}\right]=\frac{1}{2}E_{\mathrm{MTB}}\left[A\right]+\mu_{\mathrm{Mo}/W}-\frac{1}{2}E_{\mathrm{MTB}}-\mu_{A}$$

Here, *E*_MTB_[A] stands for the total energy of the system (normalized per the number of A atoms) containing a SMAC at the MTB. For this calculation, we used supercells with two A atoms to account for potential chain dimerization, as this is also observed for 8 |55 MTB without foreign atoms.^36^ The dimerization is schematically illustrated in Figure 1. *E*_MTB_ denotes the total energy of the system with the “naked” 55|8 MTB^36^ consisting of one octagon and two pentagons. μ_Mo/W_ and μ_A_ represent the chemical potentials of Mo/W and the substitutional A atom, respectively.

**Figure 1:**
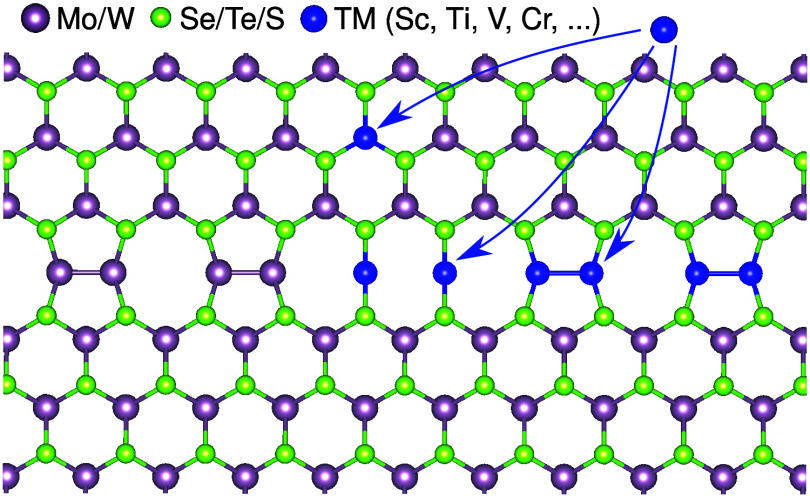
Schematic representation of MoSe_2_/MoTe_2_/WS_2_/WSe_2_ with a 55|8 mirror twin boundary, both “naked” (left) and decorated with transition metal atoms substituting for Mo/W atoms (right) and forming a single-metal-atom chain at the boundary. The chain can undergo dimerization or Peierls distortion, as illustrated on the right. A substitutional transition metal impurity in the Mo/W atom position in the basal plane is also shown.

The latter configuration, denoted “B-sub”, was calculated through

2 $$E_{f}\left[\mathrm{B‐}\mathrm{sub}\right]=\frac{1}{2}E_{\mathrm{bulk}}\left[A\right]+\mu_{\mathrm{Mo}/W}-\frac{1}{2}E_{\mathrm{bulk}}-\mu_{A}$$

Here, *E*_bulk_[A] stands for the total energy of the supercell containing a substitutional atom A at a Mo/W site (Figure 1). *E*_bulk_ is the corresponding energy of the pristine, defect-free supercell. As we are interested in the energy difference Δ*E*_f_ = *E*_f_[B-sub] – *E*_f_[C-sub], the choice of the chemical potentials for isolated atoms is not important, as these terms cancel each other.

Our theoretical approach is different from what was used by Qin et al.^40^ to describe the formation of SMACs during the chemical vapor codeposition process under S-rich conditions, where TM atoms were assumed to stick to the edges of the growing MoS_2_ islands, followed by island coalescence. SMACs can potentially also be produced by a postgrowth treatment of the systems with MTBs by TM atom deposition at elevated temperatures,^50,51^ and our methodology also describes the energetics of this direct atom exchange. We note that our approach correctly predicts the high stability of the experimentally synthesized Pt, No, Pd, and Co SMACs^39,40^ in MoS_2_.

Figure 1 shows the atomic structure of a SMAC at an MTB. The impurity atoms substitute for Mo/W atoms along the reflection line of the MTB. SMACs can undergo dimerization, a phenomenon that our calculations find to be dependent on the TM atom. We note that contrary to the free-standing chains, the periodicity of the structure is the same as the unit cell size in the host material, that is, the sum of the separations *a*, *b* between the atoms is equal to the unit cell length of the host material, e.g., *a* + *b* = *a*_MoS_2__ for MoS_2_; see also Figure S1 and Tables S1–S4.

Figure 2 presents the energy difference between C-sub and B-sub configurations for SMACs formed by various TM atoms in MoS_2_ (Figure 2b), MoSe_2_ (Figure 2c), MoTe_2_ (Figure 2d), WS_2_ (Figure 2e), and WSe_2_ (Figure 2f). SMAC formation is more likely for the TM atoms with high values of Δ*E*_f_. As mentioned above, our calculations indicate that Co, Ni, Pd, and Pt SMACs at MTBs in MoS_2_ are energetically preferable structures over isolated substitutional impurities in MoS_2_, which agrees with the experimental observations.^39,40^ The formation of SMACs is less favorable for the elements that have roughly the same number of electrons as the host Mo/W atoms, Figure 2a, as in this case, the chemical bonds in the substitutional configurations in the basal plane can be easily formed. It is also obvious that the trends cannot be explained by the atomic radii of the TM atoms, as they decrease monotonously from left to right, Figure 2a (putting aside coinage metals). Many SMACs exhibit dimerization, which correlates with their half-metal behavior to be discussed in the next section.

**Figure 2:**
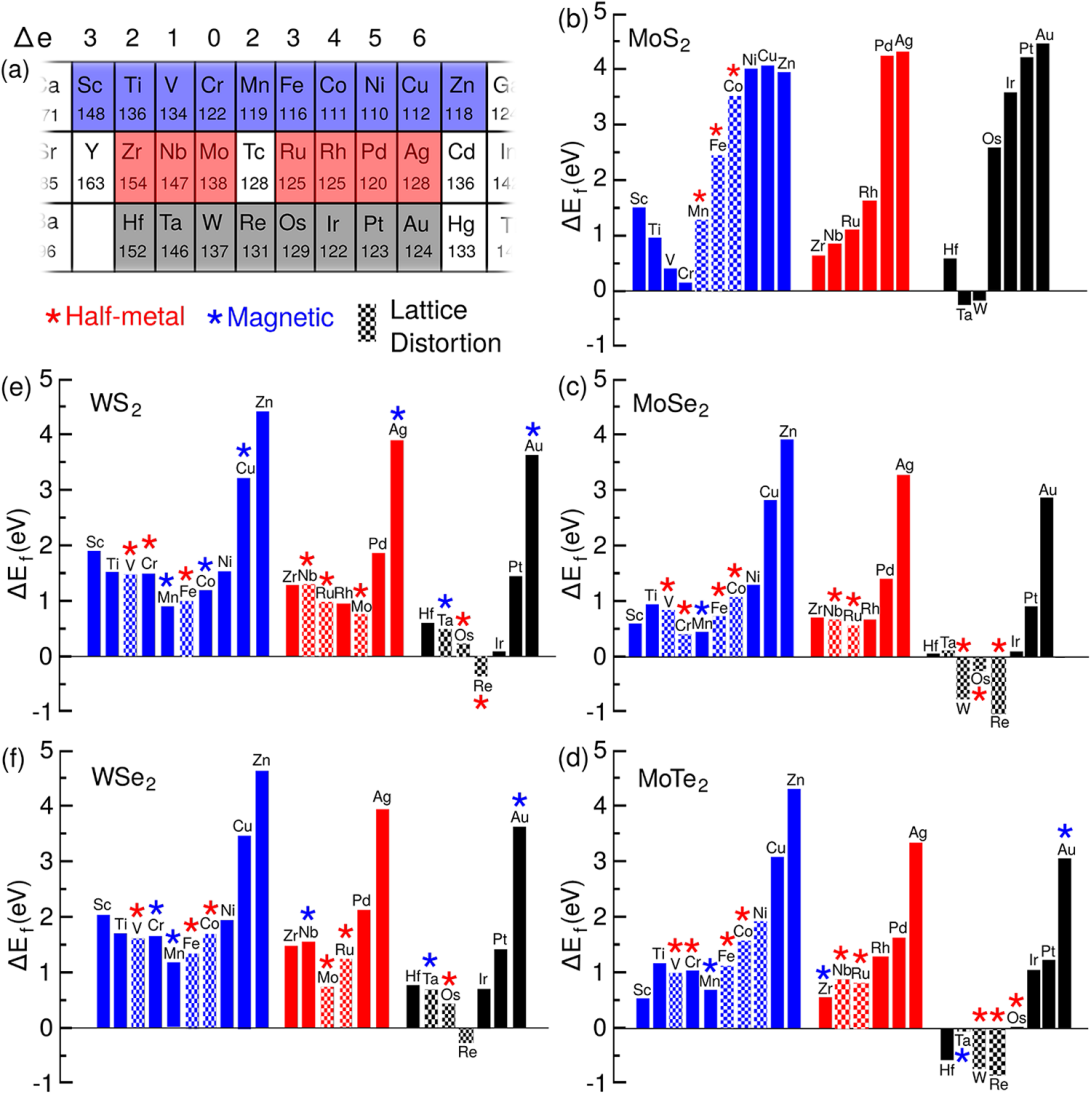
Formation energy difference Δ*E*_f_ of the C-sub and B-sub configurations in TMDs. (a) Fragment of the periodic table showing the considered transition metal atoms, their covalent radii,^52^ and the difference Δ*e* in the number of electrons in the transition metal atom and the reference Mo/W atom (top). Δ*E*_f_ for MoS_2_ (b), MoSe_2_ (c), MoTe_2_ (d), WS_2_ (e), and WSe_2_ (f). Δ*E*_f_ is positive when the C-sub configuration is energetically preferable. Half-metallic and magnetic structures are indicated with red and blue stars. Other SMACs are nonmagnetic. The structures that underwent dimerization are marked through the checkerboard pattern.

The trends are qualitatively the same for all of the TMDs we considered. However, in MoS_2_, the energy difference between the most stable nonmagnetic structures (e.g., Cu, Zn) and half-metallic SMACs (e.g., Co), the most interesting systems in the context of spintronics, is smaller than in other TMDs, but the formation of SMACs is still energetically favorable.

To assess the thermal stability of the proposed SMAC structures, we performed DFT molecular dynamics simulations for Co–MoSe_2_, selected as a representative and relatively less energetically favorable configuration. The simulations were carried out at 800 K for 4 ps. The results show that the overall structure remains intact throughout the simulation, with only minor local rearrangements occurring within the SMAC region (see Movie S1). While this simulation cannot be considered definitive proof of long-term thermodynamic stability on macroscopic time scales, it nevertheless provides strong evidence for the structural robustness of the SMAC configuration under elevated thermal conditions.

### Electronic and Magnetic Structure of Transition Metal Atom Chain at Mirror Twin Boundaries

After geometry optimization and energetics assessment, we studied the electronic structure and magnetic properties of SMACs at MTBs in MoSe_2_, MoTe_2_, WS_2_, and WSe_2_. The symmetry of TMDs^53^ prevents the construction of a periodic supercell containing an MTB, making the introduction of edges necessary. By projecting the electronic states onto atoms in the edge, bulk, and interface regions, we can investigate the effects of each region on the electronic structure. The presence of edges leads to the formation of localized edge states. A systematic study of edge effects was previously carried out^41^ by calculating the electronic structure across various edge morphologies, which showed negligible modifications of the interface states. Taking this into account, we do not show the edge states and present only the states localized at the SMACs.

All SMACs we studied exhibit metallic properties, with many of them being quasi-1D magnets (the lowest-energy spin configuration) and specifically, half-metals. As evident from Figure 2, half-metallic behavior correlates with the dimerization through the Peierls-type mechanism, which opens the gap in one spin channel. To prove this, we calculated the band structure of Co SMAC keeping the equal separation between the atoms, Figure S2. It is evident that the Fermi vector in the nonreconstructed system is at the π/2 (X) point (the Brillouin zone boundary), and the energy of the system is lowered by the gap opening. We note that we did not observe any correlation between the number of electrons in the TM atoms and the position of the Fermi energy in the chain-associated bands, Figure S8, as the chains are not free-standing, but embedded into the TMD so that the local atom arrangement and the Fermi energies are dictated by the environment. We did not observe any trimerization either (for the system composed of three primitive cells along the chain axis).

Figure 3 presents the spin-split band structure in the vicinity of the Fermi level for Co SMACs in MoS_2_ (Figure 2a), MoSe_2_ (Figure 2b), MoTe_2_ (Figure 2c), WS_2_ (Figure 2d), and WSe_2_ (Figure 2e). All systems, except for WS_2_, exhibit half-metal behavior. The gap in one of the spin channels varies between 0.1 and 0.4 eV. Figure 3f shows the spin density and atomic structure of the Co SMAC in MoSe_2_ in the ferromagnetic configuration. Dimerization of the chain is clearly visible. The typical electronic structure of a magnetic but not half-metallic system is presented in Figure S3 for Mn SMACs in MoSe_2_ and WS_2_. We note that dimerization is absent in these structures, see Tables S1 and S3, as the Fermi vector is not at the Brillouin zone boundaries, as is evident from Figure S3.

**Figure 3:**
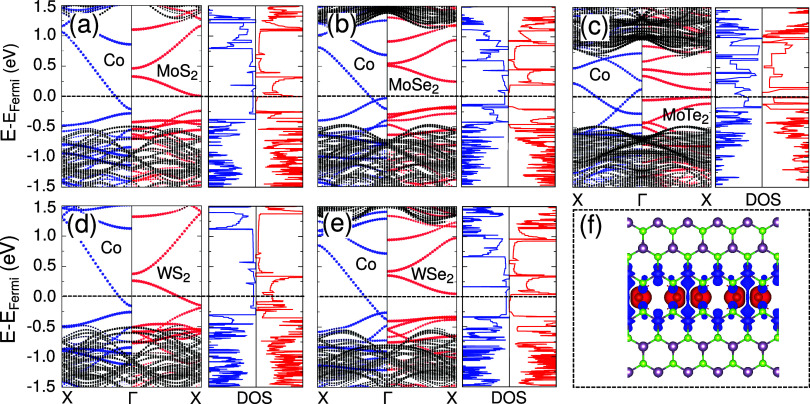
(a–e) Electronic structures of Co atoms at MTBs in different TMDs. Blue symbols represent the majority and red the minority spin states localized at the SMAC. Black stands for bulk states considering both spins. (f) Spin density in a Co chain in MoSe_2_. Here, red and blue colors stand for majority/minority spin density, and green and brown balls represent Se and Mo atoms, respectively.

For the sake of comparison, in Figure 4a–e, we show the electronic structure of nonmagnetic Zn SMAC at MTBs in TMDs. Similar to Co and other TMs, new bands in the band gap of the semiconducting host materials appear. The bands have the same shape for all TMDs, but their positions with regard to the CBM and VBM vary, as the TMDs have different band gaps. All systems are metallic but nonmagnetic, and no dimerization occurs, as evident from Figure 4f.

**Figure 4:**
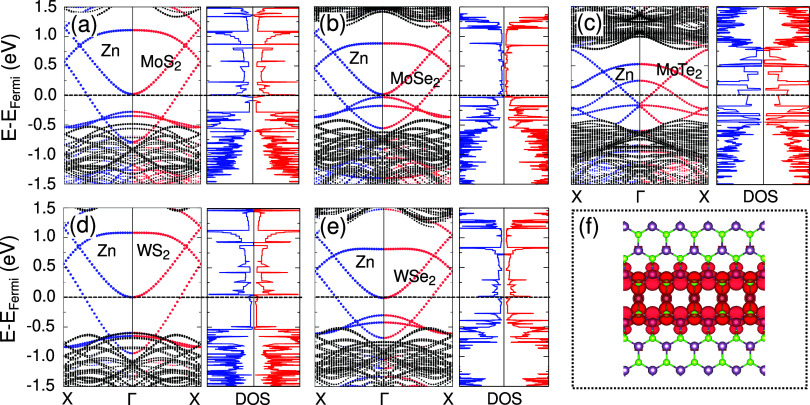
(a–e) Electronic structures of Zn SMACs at MTBs in different TMDs. Blue symbols represent up and red interface down spin states localized at the SMAC. Black symbols stand for bulk states considering both spins. (f) Electron density in the energy range corresponding to the states in the band gap of MoSe_2_.

In Figures S4–S7, we present the electronic structure for half-metallic SMACs in other TMDs we studied. The band structure and local densities of states of SMACs in MoS_2_ were previously calculated.^41^ The gap values, their positions with respect to the Fermi energy, and the widths of SMAC-associated bands strongly vary. This potentially offers many opportunities for engineering spintronic devices equipped with a back gate^54^ as small gate voltages could further tune the electronic characteristics of SMACs. In Figures S4–S7, we also show the results of noncollinear calculations of the band structures with account for SOC. No substantial changes in the band structures were observed, in agreement with the results of previous calculations for MoS_2_.^41^ We did not study the effects of the Hubbard *U* parameter on the electronic structures of SMACs (in all calculations *U* was set to zero), as although finite *U* and generally, the choice of the exchange-correlation functional, can affect the relative energy difference between the AFM/FM states of the free-standing^55^ chains or chains on metal surfaces,^56^ our previous calculations for these systems^41^ pointed out that they are small and moreover, the values of *U* are not known for these specific systems.

We also studied the magnetic properties of the half-metal systems in more detail. To evaluate the exchange interactions as schematically illustrated in Figure S1, we carried out total energy calculations for different spin configurations. We note that for the systems with strong Peierls distortion in MoS_2_, e.g., Fe, Co, we were unable to converge the system into antiferromagnetic states when magnetic moments on the adjacent atoms pointed in opposite directions,^41^ as the separation between the atoms is small and the ferromagnetic interaction is very strong: instead, we always obtained a FM or nonmagnetic solution. However, as the unit cell size of selenides and tellurides is larger, it was possible to obtain antiferromagnetic order for these systems. The energy differences between the configurations and magnetic moments on the embedded TM atoms are listed in Tables S1–S4. Magnetic moments are the largest for Cr, Mn, and Fe, that is, they correlate with the number of unpaired d-electrons. The energy difference between magnetic configurations can be used as input parameters for (semi) analytical Heisenberg-type models of magnetism in 1D systems.^57–59^

### Hydrogen Evolution Reaction for SMACs in MoSe_2_

It is not necessary to reiterate that green hydrogen production via water electrolysis plays a central role in building a sustainable energy system,^60^ but the progress in electrochemical devices for hydrogen generation through HER is largely constrained by the challenges related to the materials design and engineering of the electrode components.^61^ In that context, taking SMACs in MoSe_2_ as model systems, we assessed the catalytic properties of these systems.

For optimal catalytic performance, a material must avoid both excessively strong and excessively weak binding of reaction intermediates, as described by the Sabatier principle. The HER rate is maximized at moderate hydrogen adsorption free energy Δ*G*_H*_ values: overly positive values hinder hydrogen adsorption, while overly negative values impede desorption, causing the surface to become saturated with hydrogen.^62,63^ Introducing impurities or defects to induce structural disorder is an effective strategy for developing active, earth-abundant HER electrocatalysts by tuning Δ*G*_H*_.

Unlike typical defect structures that enhance chemical activity through the creation of unsaturated bonds, MTBs form well-ordered quasi-1D crystalline architectures without broken bonds. As a result, the surface largely preserves its van der Waals nature with its properties mainly altered by the electronic influence of the metallic MTBs. Consistent with this picture, previous theoretical studies have shown that the absence of unsaturated bonds leads to only weak interactions between water molecules and MTBs, yielding adsorption characteristics that are essentially comparable to those of a pristine, defect-free basal plane.^64^ These findings were further validated experimentally using scanning tunneling microscopy, which revealed that more complex morphologies, e.g., vertices, exhibit higher catalytic activity compared to the MTBs.^65^ To this end, SMACs offer an effective engineering strategy to enhance the catalytic performance of MTBs while preserving their intrinsic structural stability and a well-defined one-dimensional character. Moreover, the modified electronic properties introduced by SMACs improve charge transport within semiconducting TMD-based electrocatalysts, thereby accelerating interfacial electron transfer and enhancing the overall reaction kinetics.

Our H adsorption calculations show that the interstitial configuration of H atoms is the energetically most preferred configuration. The increased size of MoSe_2_ primitive cell, relative to MoS_2_, results in greater interstitial free space for hydrogen. A comparable behavioral pattern has been documented for H^66^ and transition metal atoms adsorbed on a MoTe_2_ substrate.^67^ However, in the 8 |55 MTB configuration, the hydrogen atom chooses to reside within the 8-fold ring. From Figure 5c, it is evident that Ir, Co, Pt, and Fe SMACs exhibit optimal HER activity as Δ*G*_H*_ is close to 0 eV. Here, for comparison, we also show Δ*G*_H*_ of the standard Pt(111) surface. We also observed that Δ*G*_H*_ for all SMACs is lower than that of pristine MoSe_2_ but higher than that for Se-vacancy.

**Figure 5:**
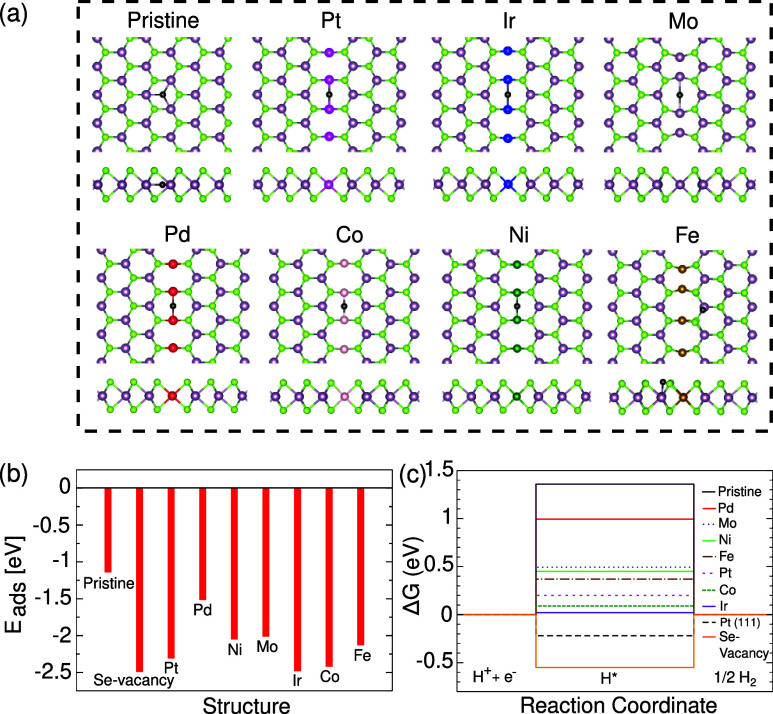
(a) Optimized atomic structures of hydrogen adsorption on SMACs in MoSe_2_ and also pristine MoSe_2_. (b) Absorption energies of H atom on SMACs and pristine MoSe_2_. (c) Free energy profiles of the HER on different SMACs in MoSe_2_ as compared to pristine (Note: the results are obtained at external potential *U*_ext_ = 0.0 V).

To gain deeper insight into the underlying chemical bonding between the SMAC and hydrogen, we analyzed the electronic structure of systems with the highest (Pd) and lowest (Ir) HER energy barriers (see Figure S9). In the case of Ir, the projected density of states (PDOS) reveals a strong overlap between H and Ir states near the Fermi level, indicating significant hybridization and the active participation of partially filled d orbitals in bonding. In contrast, for Pd, the d states show weaker overlap with the H-derived states, which are shifted to lower energies and lie farther from the Fermi level. Consequently, Ir provides more favorable hydrogen adsorption and enhanced HER activity compared with Pd.

We note that the incorporation of single metal atoms as substitutional dopants is an effective strategy to enhance hydrogen generation activity in transition metal dichalcogenides, as previously reported.^68,69^ We further compared the Gibbs free energy of hydrogen adsorption for four transition metal dopants (Mo, Fe, Co, and Ni) on TMD surfaces with those in SMAC structures (see Table S5). The results indicate that the HER energy barrier for the dopant configurations is generally higher than that of the SMAC structures. This difference can be attributed to the coordination environment: most dopant atoms remain 6-fold coordinated to Se atoms, whereas in SMACs they are typically 4-fold coordinated (or 5-fold coordinated in the case of dimerization). As a result, the greater number of unpaired electrons in SMAC structures enhances hydrogen adsorption. An exception is observed for Ni dopants, where two Ni–Se bonds are broken, resulting in a 4-fold-coordinated Ni atom.^69^

## Conclusion

To conclude, our first-principles calculations of the energetics of SMACs at MTBs in the most common TMD monolayers with the H-phase atomic structure indicate that the formation of SMACs is energetically preferable over having isolated substitutional impurities in the basal plane of the TMDs, pointing out that these structures can be synthesized, following the first successful experiments where Pt, Co, Ni, and Pd SMACs at MTBs in MoS_2_ were manufactured.^39,40^ We further studied the electronic and magnetic properties of these systems and demonstrated that many structures exhibit half-metallic electronic behavior associated with a Peierls-like distortion. SMACs can be highly beneficial in spintronic applications. Although the feasibility of half-metallic SMAC formation is lower than that of nonmagnetic ones, these systems may still be created either by CVD-type techniques, via TM atom adsorption at the edges of TMD islands followed by a second TMD growth step, or through atom exchange during postsynthesis deposition of TM atoms on the TMDs with MTB networks and mild temperature treatment. We stress that in this work, we concentrate on the energetics, which alone cannot guarantee that SMACs can be manufactured by either method. The kinetics should also be important, which requires calculations of potential barriers for different atomic processes, such as additions of Mo and S atoms at the edges of the flake passivated with impurity atoms during its CVD-type growth or atom exchange at the basal plane and at the MTB, which has previously been formed in the chalcogen-deficient material. The migration of the species on top of the TMD flake, their agglomeration, and interaction with point defects^70^ and the MTB should also be accounted for. The detailed analysis of these processes, which should be done by a combination of first-principles calculations and kinetic Monte Carlo simulations, is beyond the scope of this work. Finally, taking MoSe_2_ as the model host material, we studied the potentials of various SMACs as catalysts in the hydrogen evolution reaction. Our calculations indicate that some of the SMACs should exhibit HER activity comparable to or even superior to that of the standard Pt(111) surface.

## Supplementary Material





## Notes

Behavior of Co SMAC in MoSe_2_ at 800 K (MP4)
Details of Gibbs free energy evaluation and additional results of the calculations (PDF)

## References

[1] Peierls, R. Quantum Theory of Solids; Oxford University Press: London, 1955.

[2] Yeom H. W., Takeda S., Rotenberg E., Matsuda I., Horikoshi K., Schaefer J., Lee C. M., Kevan S. D., Ohta T., Nagao T., Hasegawa S. (1999). Instability and Charge Density Wave
of Metallic Quantum Chains on a Silicon Surface. Phys. Rev. Lett..

[3] Wang L., Wu Y., Yu Y., Chen A., Li H., Ren W., Lu S., Ding S., Yang H., Xue Q.-K., Li F.-S., Wang G. (2020). Direct Observation
of One-Dimensional Peierls-type Charge Density
Wave in Twin Boundaries of Monolayer MoTe2. ACS Nano.

[4] Deng J., Huo D., Bai Y., Lin X., Cheng Z., Zhang C. (2023). Observations
of Charge-Density-Wave States in W6Te6 Wires. Nano Lett..

[5] Yang X., Xian J.-J., Li G., Nagaosa N., Zhang W.-H., Qin L., Zhang Z.-M., Lü J.-T., Fu Y.-S. (2020). Possible Phason-Polaron
Effect on Purely One-Dimensional Charge Order of Mo_6_Se_6_ Nanowires. Phys. Rev. X.

[6] Tomonaga S.-i. (1950). Remarks
on Bloch’s Method of Sound Waves applied to Many-Fermion Problems. Prog. Theor. Phys..

[7] Luttinger J. M. (1963). An Exactly
Soluble Model of a Many-Fermion System. J. Math.
Phys..

[8] Jolie W., Murray C., Weiß P. S., Hall J., Portner F., Atodiresei N., Krasheninnikov A. V., Busse C., Komsa H.-P., Rosch A., Michely T. (2019). Tomonaga-Luttinger Liquid in a Box:
Electrons Confined within MoS_2_ Mirror-Twin Boundaries. Phys. Rev. X.

[9] Stühler R., Reis F., Müller T., Helbig T., Schwemmer T., Thomale R., Schäfer J., Claessen R. (2020). Tomonaga-Luttinger
liquid in the edge channels of a quantum spin Hall insulator. Nat. Phys..

[10] Zhu T., Ruan W., Wang Y.-Q., Tsai H.-Z., Wang S., Zhang C., Wang T., Liou F., Watanabe K., Taniguchi T., Neaton J. B., Weber-Bargioni A., Zettl A., Qiu Z. Q., Zhang G., Wang F., Moore J. E., Crommie M. F. (2022). Imaging gate-tunable Tomonaga-Luttinger
liquids in 1H-MoSe2 mirror twin boundaries. Nat. Mater..

[11] Nadj-Perge S., Drozdov I. K., Li J., Chen H., Jeon S., Seo J., MacDonald A. H., Bernevig B. A., Yazdani A. (2014). Observation of Majorana
fermions in ferromagnetic atomic chains on a superconductor. Science.

[12] Schneider L., Beck P., Neuhaus-Steinmetz J., Rózsa L., Posske T., Wiebe J., Wiesendanger R. (2022). Precursors
of Majorana modes and their length-dependent energy oscillations probed
at both ends of atomic Shiba chains. Nat. Nanotechnol..

[13] Choi D.-J., Lorente N., Wiebe J., von Bergmann K., Otte A. F., Heinrich A. J. (2019). Colloquium: Atomic
spin chains on
surfaces. Rev. Mod. Phys..

[14] Steinbrecher M., Rausch R., That K. T., Hermenau J., Khajetoorians A. A., Potthoff M., Wiesendanger R., Wiebe J. (2018). Non-collinear spin
states in bottom-up fabricated atomic chains. Nat. Commun..

[15] Schneider L., Beck P., Posske T., Crawford D., Mascot E., Rachel S., Wiesendanger R., J Wiebe. (2021). Topological Shiba bands in artificial
spin chains on superconductors. Nat. Phys..

[16] Gambardella P., Dallmeyer A., Maiti K., Malagoli M. C., Eberhardt W., Kern K., Carbone C. (2002). Ferromagnetism in one-dimensional
monatomic metal chains. Nature.

[17] Pickett W. E., Eschrig H. (2007). Half metals: from formal
theory to real material issues. J. Phys.:Condens.
Matter.

[18] Crain J. N., Pierce D. T. (2005). End States in One-Dimensional
Atom Chains. Science.

[19] Chen C., Bobisch C. A., Ho W. (2009). Visualization
of Fermi’s Golden
Rule Through Imaging of Light Emission from Atomic Silver Chains. Science.

[20] Liu M., Artyukhov V. I., Yakobson B. I. (2017). Mechanochemistry of One-Dimensional
Boron: Structural and Electronic Transitions. J. Am. Chem. Soc..

[21] Baibich M. N., Broto J. M., Fert A., Van Dau F. N., Petroff F., Etienne P., Creuzet G., Friederich A., Chazelas J. (1988). Giant Magnetoresistance of (001)­Fe/(001)Cr Magnetic
Superlattices. Phys. Rev. Lett..

[22] Parkin S., Jiang X., Kaiser C., Panchula A., Roche K., Samant M. (2003). Magnetically engineered
spintronic sensors and memory. Proc. IEEE.

[23] Pearton S. J., Norton D. P., Frazier R., Han S. Y., Abernathy C. R., Zavada J. M. (2005). Spintronics Device
Concepts. IEE Proc-Circuits Devices Syst..

[24] Yanson A. I., Bollinger G. R., van den Brom H. E., Agrat N., van Ruitenbeek J. M. (1998). Formation
and manipulation of a metallic wire of single gold atoms. Nature.

[25] Sokolov A., Zhang C., Tsymbal E. Y., Redepenning J., Doudin B. (2007). Quantized magnetoresistance in atomic-size
contacts. Nat. Nanotechnol..

[26] Calvo M. R., Fernández-Rossier J., Palacios J. J., Jacob D., Natelson D., Untiedt C. (2009). The Kondo
effect in ferromagnetic
atomic contacts. Nature.

[27] Snijders P. C., Weitering H. H. (2010). Colloquium:
Electronic instabilities in self-assembled
atom wires. Rev. Mod. Phys..

[28] Elibol K., Susi T., Mangler C., Eder D., Meyer J. C., Kotakoski J., Hobbs R. G., van Aken P. A., Bayer B. C. (2023). Linear
indium atom chains at graphene edges. npj 2D
Mater. Appl..

[29] Senga R., Komsa H.-P., Liu Z., Hirose-Takai K., Krasheninnikov A. V., Suenaga K. (2014). Atomic structure and
dynamic behaviour
of trulyone-dimensional ionic chains inside carbonnanotubes. Nat. Mater..

[30] Fujimori T., Morelos-Gómez A., Zhu Z., Muramatsu H., Futamura R., Urita K., Terrones M., Hayashi T., Endo M., Young Hong S., Chul Choi Y., Tománek D., Kaneko K. (2013). Conducting linear chains of sulphur
inside carbon nanotubes. Nat. Commun..

[31] Komsa H.-P., Senga R., Suenaga K., Krasheninnikov A. V. (2017). Structural
Distortions and Charge Density Waves in Iodine Chains Encapsulated
inside Carbon Nanotubes. Nano Lett..

[32] Bylinskii A., Gangloff D., Counts I., Vuletić V. (2016). Observation
of Aubry-type transition in finite atom chains via friction. Nat. Mater..

[33] Jorio, A. ; Dresselhaus, G. ; Dresselhaus, M. Carbon Nanotubes: Advanced Topics in the Synthesis, Structure, Properties and Applications; Springer, 2008.

[34] Ma M., Guo S., Sang X., Gao C., Liu Z., He Y. (2022). Structure,
synthesis, and properties of single-metal-atom chains. Cell Rep. Phys. Sci..

[35] Ávalos
Ovando O., Mastrogiuseppe D., Ulloa S. E. (2019). Lateral heterostructures
and one-dimensional interfaces in 2D transition metal dichalcogenides. J. Phys.:Condens. Matter.

[36] Komsa H.-P., Krasheninnikov A. V. (2017). Engineering
the Electronic Properties of Two-Dimensional
Transition Metal Dichalcogenides by Introducing Mirror Twin Boundaries. Adv. Electron. Mater..

[37] Yang X., Gu Z.-L., Wang H., Xian J.-J., Meng S., Nagaosa N., Zhang W.-H., Liu H.-W., Ling Z.-H., Fan K., Zhang Z.-M., Qin L., Zhang Z.-H., Liang Y., Li J.-X., Fu Y.-S. (2023). Manipulating
Hubbard-type Coulomb
blockade effect of metallic wires embedded in an insulator. Natl. Sci. Rev..

[38] van
Efferen C., Fischer J., Costi T. A., Rosch A., Michely T., Jolie W. (2024). Modulated Kondo screening along magnetic
mirror twin boundaries in monolayer MoS2. Nat.
Phys..

[39] Guo S., Fu J., Zhang P., Zhu C., Yao H., Xu M., An B., Wang X., Tang B., Deng Y., Salim T., Du H., Dunin-Borkowski R. E., Xu M., Zhou W., Tay B. K., He Y., Hofmann M., Hsieh Y.-P., Guo W. (2022). Direct growth of single-metal-atom chains. Nat. Synth..

[40] Qin W., Guo S., Liu Z., Zhang P., Zhu C., Wu Y., Qiao R., Liu Z., Guo W., Zhang Z. (2025). Coherently
confined single-metal-atom chains in 2D semiconductors. Nat. Commun..

[41] Davies F. H., Krasheninnikov A. V. (2024). Chains
of atoms embedded into transition metal dichalcogenides
as one-dimensional half-metallic magnets. Phys.
Rev. B.

[42] Han S. A., Bhatia R., Kim S.-W. (2015). Synthesis, properties and potential
applications of two-dimensional transition metal dichalcogenides. Nano Convergence.

[43] Choi W., Choudhary N., Han G. H., Park J., Akinwande D., Lee Y. H. (2017). Recent development of two-dimensional transition metal
dichalcogenides and their applications. Mater.
Today.

[44] Manzeli S., Ovchinnikov D., Pasquier D., Yazyev O. V., Kis A. (2017). 2D transition
metal dichalcogenides. Nat. Rev. Mater..

[45] Fu Q., Han J., Wang X., Xu P., Yao T., Zhong J., Zhong W., Liu S., Gao T., Zhang Z., Xu L., Song B. (2021). 2D Transition Metal
Dichalcogenides: Design, Modulation,
and Challenges in Electrocatalysis. Adv. Mater..

[46] Kresse G., Furthmüller J. (1996). Efficiency of ab-initio total energy
calculations for
metals and semiconductors using a plane-wave basis set. Comput. Mater. Sci..

[47] Perdew J. P., Burke K., Ernzerhof M. (1996). Generalized
Gradient Approximation
Made Simple. Phys. Rev. Lett..

[48] Kresse G., Joubert D. (1999). From ultrasoft pseudopotentials
to the projector augmented-wave
method. Phys. Rev. B.

[49] Pack J. D., Monkhorst H. J. (1977). Special
points for Brillouin-zone integrations”–a
reply. Phys. Rev. B.

[50] Coelho P. M., Komsa H.-P., Coy Diaz H., Ma Y., Krasheninnikov A. V., Batzill M. (2018). Post-Synthesis Modifications
of Two-Dimensional MoSe_2_or MoTe_2_ by Incorporation
of Excess Metal Atoms
into the Crystal Structure. ACS Nano.

[51] Coelho P. M., Komsa H.-P., Lasek K., Kalappattil V., Karthikeyan J., Phan M.-H., Krasheninnikov A. V., Batzill M. (2019). Room temperature ferromagnetism in MoTe_2_ by post-growth incorporation of vanadium impurities. Adv. Electron. Mater..

[52] https://sciencenotes.org/covalent-radius-definition-and-trend/.

[53] Ribeiro-Soares J., Almeida R. M., Barros E. B., Araujo P. T., Dresselhaus M. S., Cançado L. G., Jorio A. (2014). Group theory analysis of phonons
in two-dimensional transition metal dichalcogenides. Phys. Rev. B.

[54] Ahn H., Moon G., Jung H.-g., Deng B., Yang D. H., Yang S., Han C., Cho H., Yeo Y., Kim C.-J., Yang C.-H., Kim J., Choi S.-Y., Park H., Jeon J., Park J.-H., Jo M.-H. (2024). Integrated
1D epitaxial mirror twin boundaries for ultrascaled 2D MoS_2_ field-effect transistors. Nat. Nanotechnol..

[55] Amini J., Alaei M., de Gironcoli S. (2025). 1D transition
metal oxide chains
as a challenging model for abinitio calculations. J. Chem. Phys..

[56] Mokrousov Y., Bihlmayer G., Blügel S., Heinze S. (2007). Magnetic order and
exchange interactions in monoatomic 3d transition-metal chains. Phys. Rev. B.

[57] Curilef S., del Pino L. A., Orellana P. (2005). Ferromagnetism in one
dimension:
Critical temperature. Phys. Rev. B.

[58] Xue Y., Shen Z., Wu Z., Song C. (2022). Theoretical prediction
of Curie temperature in two-dimensional ferromagnetic monolayer. J. Appl. Phys..

[59] Xiang H., Lee C., Koo H.-J., Gong X., Whangbo M.-H. (2013). Magnetic properties
and energy-mapping analysis. Dalton Trans..

[60] Muradov N., Veziroglu T. (2005). From hydrocarbon
to hydrogen-carbon to hydrogen economy. Int.
J. Hydrogen Energy.

[61] Li L., Wu Z., Yuan S., Zhang X.-B. (2014). Advances and challenges for flexible
energy storage and conversion devices and systems. Energy Environ. Sci..

[62] Ekspong J., Gracia-Espino E., WÅgberg T. (2020). Hydrogen Evolution Reaction Activity
of Heterogeneous Materials: A Theoretical Model. J. Phys. Chem. C.

[63] Parsons R. (1958). The rate of
electrolytic hydrogen evolution and the heat of adsorption of hydrogen. Trans. Faraday Soc..

[64] Li J., Joseph T., Ghorbani-Asl M., Kolekar S., Krasheninnikov A. V., Batzill M. (2021). Mirror twin boundaries in MoSe_2_ monolayers
as one dimensional nanotemplates for selective water adsorption. Nanoscale.

[65] Lunardon M., Kosmala T., Ghorbani-Asl M., Krasheninnikov A. V., Kolekar S., Durante C., Batzill M., Agnoli S., Granozzi G. (2023). Catalytic Activity of Defect-Engineered Transition
Me tal Dichalcogenides Mapped with Atomic-Scale Precision by Electrochemical
Scanning Tunneling Microscopy. ACS Energy Lett..

[66] Kosmala T., Coy Diaz H., Komsa H.-P., Ma Y., Krasheninnikov A. V., Batzill M., Agnoli S. (2018). Metallic Twin
Boundaries Boost the
Hydrogen Evolution Reaction on the Basal Plane of Molybdenum Selenotellurides. Adv. Energy Mater..

[67] Karthikeyan J., Komsa H.-P., Batzill M., Krasheninnikov A. V. (2019). Which Transition
Metal Atoms Can Be Embedded into Two-Dimensional Molybdenum Dichalcogenides
and Add Magnetism?. Nano Lett..

[68] Li S., Luo Z., Wang S., Cheng H. (2023). Atomic structure and HER performance
of doped MoS_2_: A mini-review. Electrochem.
Commun..

[69] Jain A., Sadan M. B., Ramasubramaniam A. (2020). Promoting Active Sites for Hydrogen
Evolution in MoSe_2_ via Transition-Metal Doping. J. Phys. Chem. C.

[70] Yoshimura A., Koratkar N., Meunier V. (2020). Substitutional transition
metal doping
in MoS_2_: a first-principles study. Nano Express.

