# Supplementary material for: Single-Metal-Atom Chains at Mirror Twin Boundaries in Transition Metal Dichalcogenides: Electronic, Magnetic, and Catalytic Properties
Source: ACS Appl Mater Interfaces. 2026 Jun 4;18(23):32822–9. doi: 10.1021/acsami.6c03473 (PMC13288380; doi:10.1021/acsami.6c03473)
Supplement: Supplementary file 2 [file am6c03473_si_002.pdf]

Supporting information to "Single-Metal-Atom Chains at Mirror Twin Boundaries in Transition-Metal Dichalcogenides: Electronic, Magnetic, and Catalytic Properties"

Prosun Santra,[†] Mahdi Ghorbani-Asl,[†] Wouter Jolie,[‡] and Arkady V. Krasheninnikov[*,†]

†*Institute of Ion Beam Physics and Materials Research, Helmholtz-Zentrum Dresden-Rossendorf, 01328 Dresden, Germany*

‡*II. Physikalisches Institut, Universität zu Köln, Zülpicher Straße 77, 50937 Köln, Germany*

E-mail: a.krasheninnikov@hzdr.de

Gibbs free energy calculations

The Gibbs free energy of hydrogen adsorption (ΔG_{H^*}) was calculated as follows:

$$\Delta G_{\mathrm{H}}* = \Delta E_{\mathrm{H}} * + \Delta E_{\mathrm{ZPE}} - T\Delta S_{\mathrm{H}}* \tag{1}$$

where T is temperature, and ΔE_H*, ΔE_{ZPE}, and ΔS_H* represent the differences in energy, zero-point energy, and entropy, respectively, between a hydrogen atom adsorbed on a catalytic atom and a hydrogen molecule in the gas phase. The adsorption of hydrogen atoms onto the catalytic surface induces, in most cases, a negligible change in the entropy of the catalytic materials. Under the assumption that the entropy change ($\Delta S_{H_2}*$) is similar to -(1/2) ΔS_H*, the $T\Delta S_H*$ term in Equation 1 is estimated to be –0.24 eV at room temperature. Therefore, an estimate of ΔS_H* can be obtained from ΔE_H*. Also the adsorption energy (E_{ads}) for the adsorbate on the surface is calculated as,

$$E_{\mathrm{ads}} = E_{\mathrm{slab+adsorbate}} - (E_{\mathrm{slab}} - E_{\mathrm{adsorbate}}) \tag{2}$$

In this expression, the first term corresponds to the total energy of the slab with the adsorbate, while the second and third terms represent the energies of the isolated slab and the isolated adsorbate, respectively.

Additional results

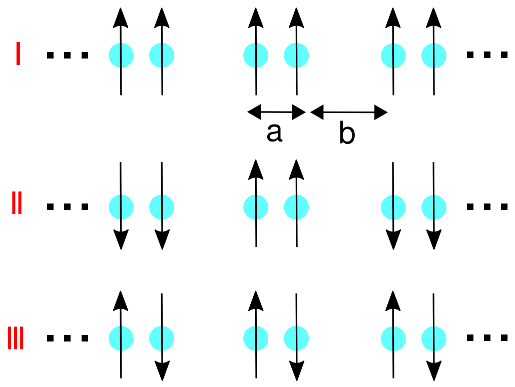

Figure S 1: Schematic illustration of spin orientations and Peierls distortion in the chains of trnasition metal atoms at the MTB.

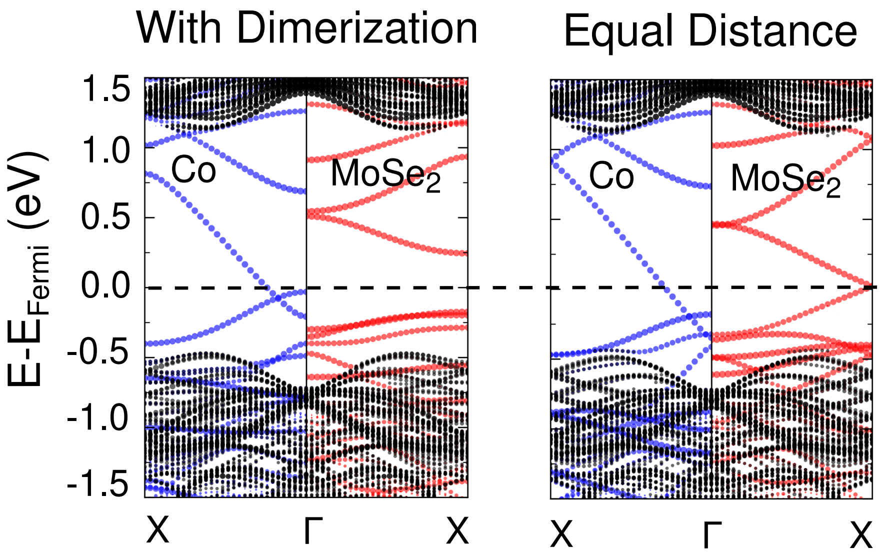

Figure S 2: Electronic structures of Co SMAC in $MoSe_2$ with and without dimerization. Blue represents interface up and red represents interface down spin states. Black stands for bulk states considering both spins.

Table S 1: Properties of embedded TM chains in $MoSe_2$ including atom separations a and b (Fig. 1) , energy differences between magnetic states and magnetic moments (M) in (μ_B) on metal atoms. The ferromagnetic state is taken as the the reference (zero) energy. Symbol – indicates that the state could not be realized in the calculations, x not calculated, as the structure was not magnetic.

	a (Å)	b (Å)	$(b-a)/(b+a)$	$E_{\uparrow\uparrow\uparrow\uparrow}$ (eV)	$E_{\uparrow\uparrow\downarrow\downarrow}$ (eV)	$E_{\uparrow\downarrow\uparrow\downarrow}$ (eV)	M (μ_B)
Sc	3.318	3.318	0.00	x	x	x	0.00
Ti	3.318	3.318	0.00	x	x	x	0.00
V	2.363	4.273	0.28	0	0.38	0.39	0.97
Cr	1.956	4.680	0.41	0	–	–	0.09
Mn	3.318	3.318	0.00	x	x	x	3.06
Fe	2.395	4.241	0.27	0	1.20	1.04	1.87
Co	2.839	3.797	0.14	0	0.20	0.20	0.73
Ni	3.317	3.319	0.00	x	x	x	0.00
Cu	3.270	3.366	0.01	x	x	x	0.00
Zn	3.315	3.321	0.00	x	x	x	0.00
Zr	3.318	3.318	0.00	x	x	x	0.00
Nb	2.618	4.018	0.21	0	–	–	0.65
Ru	2.493	4.143	0.25	0	0.07	0.08	0.48
Rh	3.318	3.318	0.00	x	x	x	0.00
Pd	3.318	3.318	0.00	x	x	x	0.00
Ag	3.294	3.342	0.01	x	x	x	0.00
Hf	3.318	3.318	0.00	x	x	x	0.00
Ta	2.821	3.815	0.15	x	x	x	0.00
W	2.343	4.293	0.29	0	0.01	0.03	0.05
Os	2.491	4.145	0.24	0	0.14	–	0.41
Re	2.309	4.327	0.30	0	–	–	0.32
Ir	3.085	3.551	0.30	x	x	x	0.00
Pt	3.318	3.318	0.00	x	x	x	0.00
Au	3.318	3.318	0.00	x	x	x	0.00

Table S 2: Properties of embedded TM chains in $MoTe_2$ including atom separations a and b (Fig. 1) , energy differences between magnetic states and magnetic moments (M) in (μ_B) on metal atoms. The ferromagnetic state is taken as the the reference (zero) energy. Symbol – indicates that the state could not be realized in the calculations, x not calculated, as the structure was not magnetic.

	a (Å)	b (Å)	$(b-a)/(b+a)$	$E_{\uparrow\uparrow\uparrow\uparrow}$ (eV)	$E_{\uparrow\uparrow\downarrow\downarrow}$ (eV)	$E_{\uparrow\downarrow\uparrow\downarrow}$ (eV)	M (μ_B)
Sc	3.549	3.549	0.00	x	x	x	0.00
Ti	3.549	3.549	0.00	x	x	x	0.00
V	2.296	4.802	0.35	0	0.03	0.03	0.89
Cr	3.549	3.549	0.00	0	0.53	0.80	2.80
Mn	3.549	3.549	0.00	x	x	x	3.22
Fe	2.311	4.786	0.35	0	0.88	0.87	1.92
Co	2.532	4.566	0.29	0	0.15	0.15	0.92
Ni	2.938	4.159	0.17	x	x	x	0.00
Cu	3.549	3.549	0.00	x	x	x	0.00
Zn	2.557	4.540	0.27	x	x	x	0.00
Zr	3.551	3.553	0.00	x	x	x	0.11
Nb	2.597	4.501	0.27	0	0.04	0.01	0.62
Ru	2.296	4.801	0.35	0	–	–	0.14
Rh	3.543	3.555	0.00	x	x	x	0.00
Pd	3.549	3.549	0.00	x	x	x	0.00
Ag	3.540	3.557	0.00	x	x	x	0.00
Hf	3.549	3.549	0.00	x	x	x	0.00
Ta	2.599	4.498	0.00	x	x	x	0.26
W	2.333	4.764	0.34	0	0.01	–	0.02
Re	2.285	4.813	0.36	0	–	0.01	0.21
Os	2.296	4.802	0.35	0	0.01	–	0.09
Ir	3.549	3.549	0.00	x	x	x	0.00
Pt	3.549	3.549	0.00	x	x	x	0.00
Au	3.549	3.549	0.00	x	x	x	0.03

Table S 3: Properties of embedded TM chains in WS_2 including atom separations a and b (Fig. 1) , energy differences between magnetic states and magnetic moments (M) in (μ_B) on metal atoms. The ferromagnetic state is taken as the the reference (zero) energy. Symbol – indicates that the state could not be realized in the calculations, x not calculated, as the structure was not magnetic.

	a (Å)	b (Å)	$(b-a)/(b+a)$	$E_{\uparrow\uparrow\uparrow\uparrow}$ (eV)	$E_{\uparrow\uparrow\downarrow\downarrow}$ (eV)	$E_{\uparrow\downarrow\uparrow\downarrow}$ (eV)	M (μ_B)
Sc	3.181	3.181	0.00	x	x	x	0.46
Ti	3.181	3.181	0.00	x	x	x	0.00
V	2.377	3.984	0.25	0	0.39	0.36	0.94
Cr	3.090	3.260	0.03	0	1.18	1.17	2.23
Mn	3.176	3.185	0.00	x	x	x	2.78
Fe	2.452	3.909	0.23	0	–	1.09	1.76
Co	3.143	3.218	0.01	x	x	x	0.50
Ni	3.178	3.183	0.00	x	x	x	0.00
Cu	3.181	3.181	0.00	x	x	x	0.28
Zn	3.176	3.185	0.00	x	x	x	0.00
Zr	3.181	3.181	0.00	x	x	x	0.00
Nb	2.700	3.660	0.15	0	–	0.29	0.51
Ru	2.540	3.820	0.20	0	0.18	0.18	0.54
Rh	3.173	3.188	0.00	x	x	x	0.00
Mo	2.250	4.110	0.29	0	0.01	–	0.05
Pd	3.181	3.181	0.00	x	x	x	0.00
Ag	3.181	3.181	0.00	x	x	x	0.14
Hf	3.181	3.181	0.00	x	x	x	0.00
Ta	2.902	3.459	0.08	x	x	x	0.07
Os	2.543	3.818	0.20	0	0.10	0.10	0.48
Re	2.339	4.022	0.26	0	–	–	0.24
Ir	3.181	3.181	0.00	x	x	x	0.00
Pt	3.181	3.181	0.00	x	x	x	0.00
Au	3.181	3.181	0.00	x	x	x	0.24

Table S 4: Properties of embedded TM chains in WSe_2 including atom separations a and b (Fig. 1), energy differences between magnetic states and magnetic moments (M) in (μ_B) on metal atoms. – not realized state, x not calculated

	a (Å)	b (Å)	$(b-a)/(b+a)$	$E_{\uparrow\uparrow\uparrow\uparrow}$ (eV)	$E_{\uparrow\uparrow\downarrow\downarrow}$ (eV)	$E_{\uparrow\downarrow\uparrow\downarrow}$ (eV)	M (μ_B)
Sc	3.316	3.316	0.00	x	x	x	0.00
Ti	3.316	3.316	0.00	x	x	x	0.00
V	2.348	4.284	0.29	0	0.08	0.11	0.95
Cr	3.297	3.335	0.00	x	x	x	2.47
Mn	3.302	3.331	0.00	x	x	x	2.96
Fe	2.396	4.236	0.28	0	1.18	–	1.90
Co	2.901	3.732	0.13	0	0.05	0.06	0.76
Ni	3.316	3.316	0.00	x	x	x	0.00
Cu	3.316	3.316	0.00	x	x	x	0.00
Zn	3.316	3.316	0.00	x	x	x	0.00
Zr	3.316	3.316	0.00	x	x	x	0.00
Nb	3.316	3.316	0.00	x	x	x	0.21
Mo	2.224	4.409	0.33	0	–	0.01	0.04
Ru	2.511	4.122	0.24	0	0.21	–	0.56
Pd	3.312	3.321	0.00	x	x	x	0.21
Ag	3.316	3.316	0.00	x	x	x	0.00
Hf	3.316	3.316	0.00	x	x	x	0.00
Ta	2.836	3.796	0.14	x	x	x	0.07
Os	2.511	4.121	0.24	0	0.10	0.10	0.48
Re	2.317	4.316	0.24	x	x	x	0.00
Ir	3.316	3.316	0.00	x	x	x	0.00
Au	3.316	3.316	0.00	x	x	x	0.04

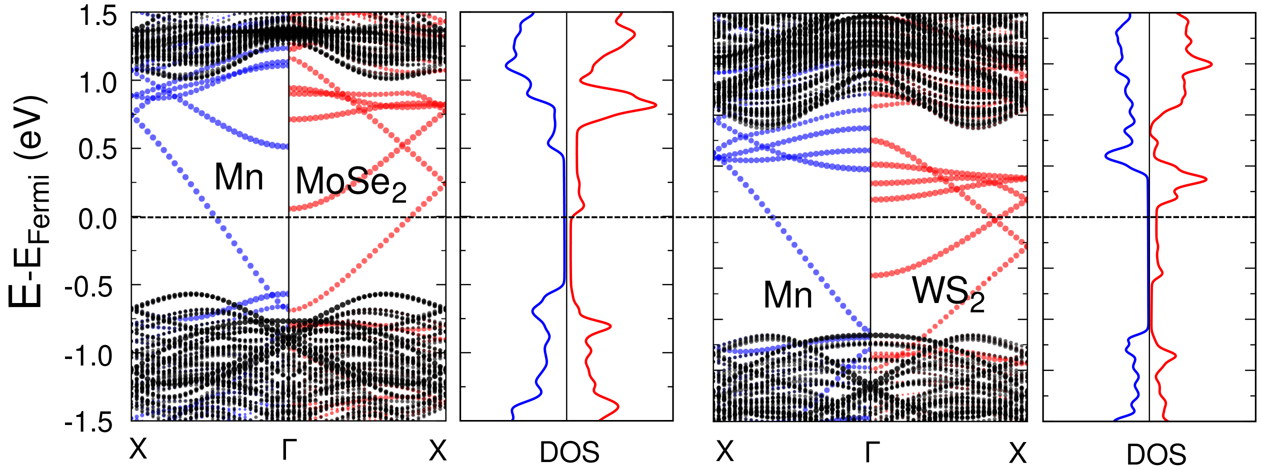

Figure S 3: Electronic structures of Mn at MTBs in different TMDs e.g. $MoSe_2$ and WS_2. Blue symbols represents up and red down spin states localized at the SMAC. Black stands for bulk states considering both spins.

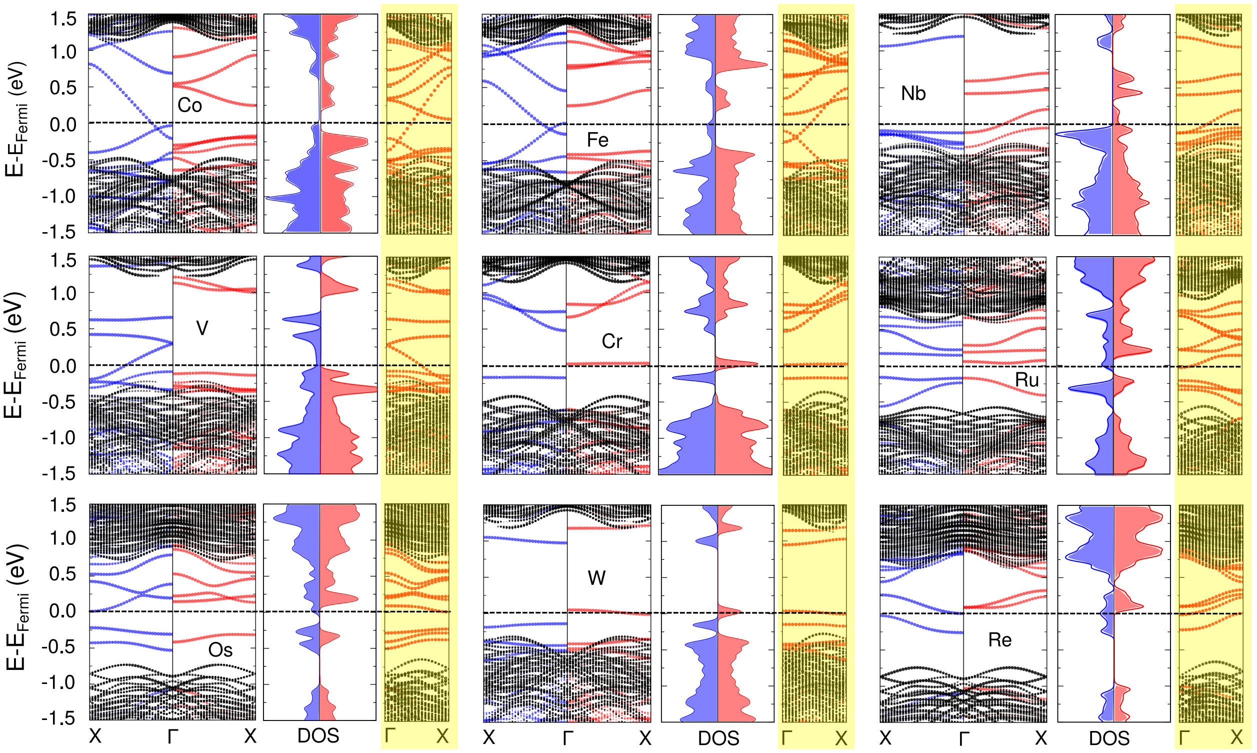

Figure S 4: Electronic structures of half metals in $MoSe_2$. Blue represents interface up and red represents interface down spin states. Black stands for bulk states considering both spins. 3rd column is band structure with SOC for each panel. The band structures calculated with account for the spin-orbit coupling are shown on yellow background.

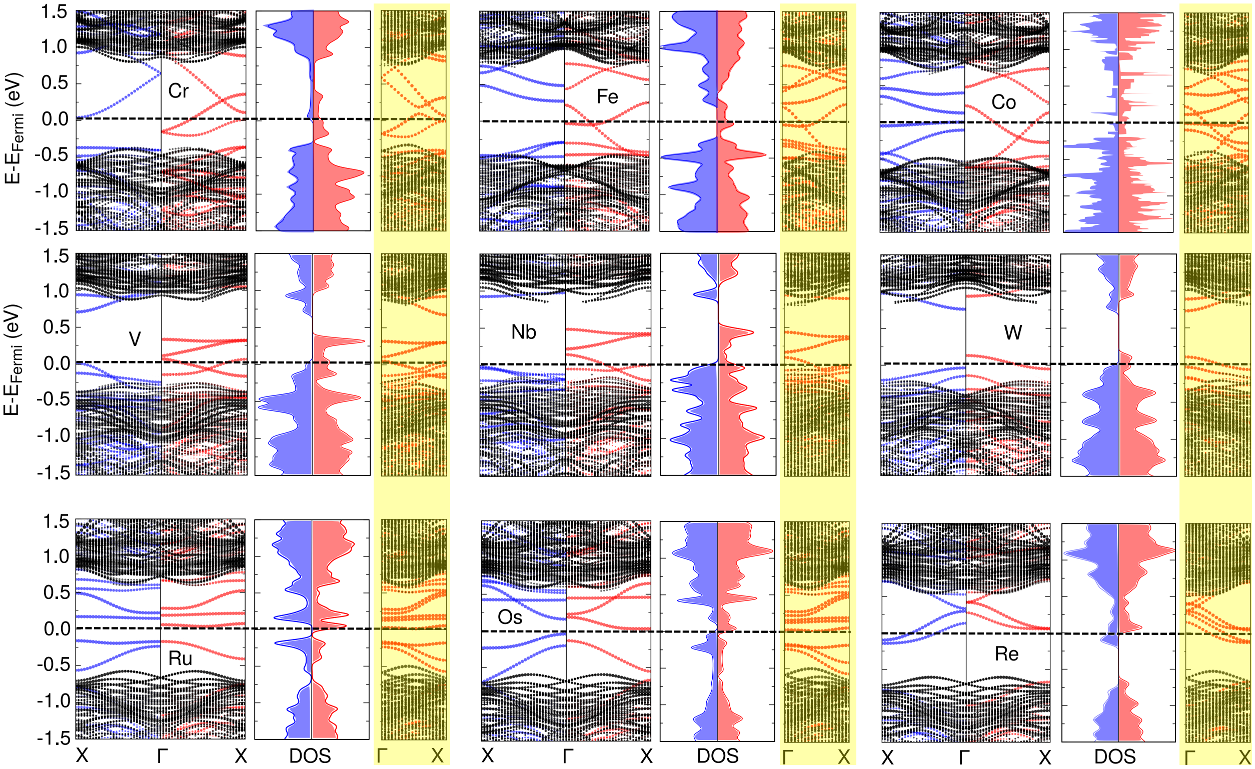

Figure S 5: Electronic structures of half metals in $MoTe_2$. Blue represents interface up and red represents interface down spin states. Black stands for bulk states considering both spins. 3rd column is band structure with SOC for each panel. The band structures calculated with account for the spin-orbit coupling are shown on yellow background.

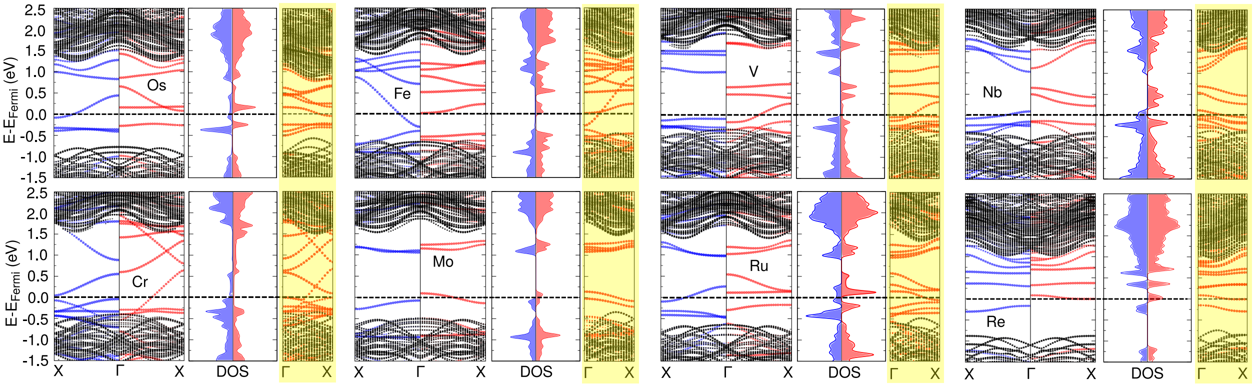

Figure S 6: Electronic structures of half metals in WS_2. Blue represents interface up and red represents interface down spin states. Black stands for bulk states considering both spins. 3rd column is band structure with SOC for each panel. The band structures calculated with account for the spin-orbit coupling are shown on yellow background.

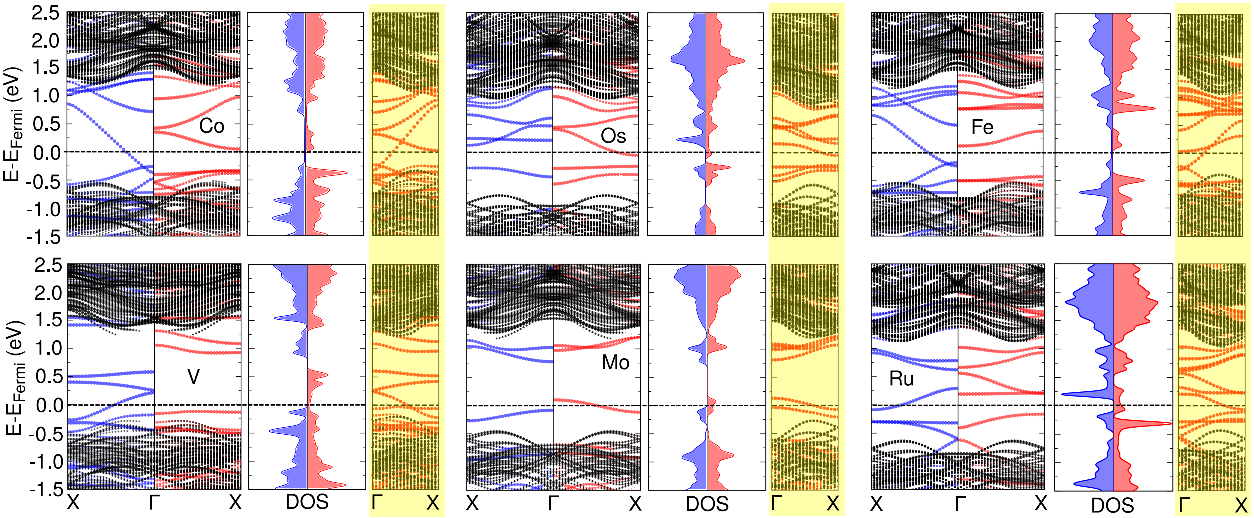

Figure S 7: Electronic structures of half metals in WSe_2. Blue represents interface up and red represents interface down spin states. Black stands for bulk states considering both spins. 3rd column is band structure with SOC for each panel. The band structures calculated with account for the spin-orbit coupling are shown on yellow background.

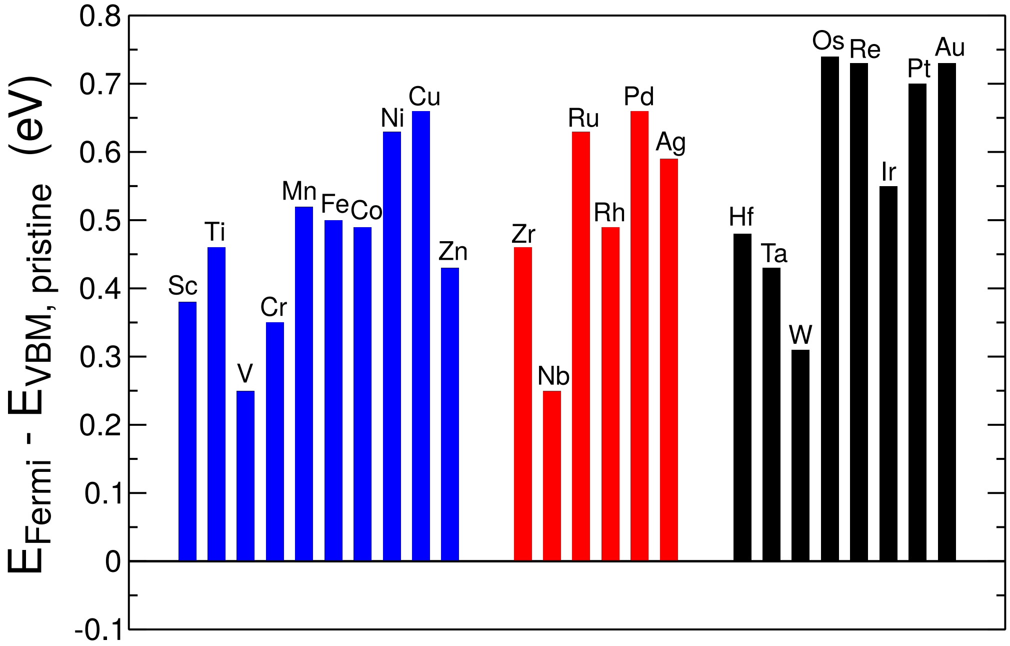

Figure S 8: Fermi energies for various SMACs in $MoSe_2$. The position of the valance band maximum in $MoSe_2$ is taken as zero energy.

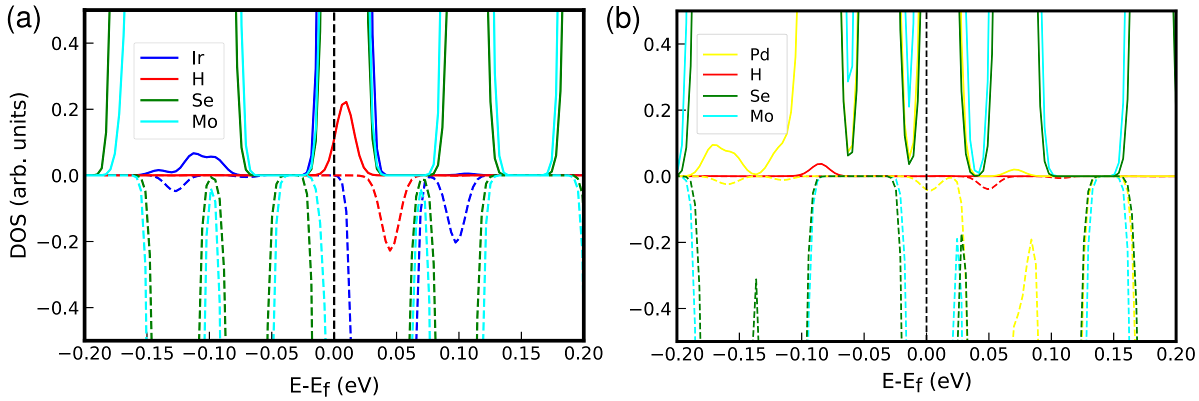

Figure S 9: Projected density of states (DOS) of SMACs formed by (a) Ir and (b) Pd in $MoSe_2$ with adsorbed H atoms. The Fermi level is shifted to zero.

Table S 5: Gibbs free energy for hydrogen evolution reaction (ΔG_H) on $MoSe_2$ with different transition metals as impurities or SMACs.

**Elements**	**ΔG_H (eV)**	
	**dopants**[1]	**SMACs**
Mo	2.00	0.49
Fe	0.80	0.37
Co	0.25	0.08
Ni	-0.20	0.45

References

1. Jain, A.; Sadan, M. B.; Ramasubramaniam, A. Promoting Active Sites for Hydrogen Evolution in MoSe2 via Transition-Metal Doping. *The Journal of Physical Chemistry C* **2020**, *124*, 12324–12336.
